# Major Depression Detection from EEG Signals Using Kernel Eigen-Filter-Bank Common Spatial Patterns

**DOI:** 10.3390/s17061385

**Published:** 2017-06-14

**Authors:** Shih-Cheng Liao, Chien-Te Wu, Hao-Chuan Huang, Wei-Teng Cheng, Yi-Hung Liu

**Affiliations:** 1 Department of Psychiatry, National Taiwan University Hospital, Taipei 10051, Taiwan; scliao@ntu.edu.tw (S.-C.L.); chientewu@ntu.edu.tw (C.-T.W.); 2 School of Occupational Therapy, College of Medicine, National Taiwan University, Taipei 10051, Taiwan; 3 Graduate Institute of Mechatronics Engineering, National Taipei University of Technology, Taipei 10608, Taiwan; alexhuang79@gmail.com; 4 Department of Mechanical Engineering, Chung Yuan Christian University, Chungli 32023, Taiwan; eric.cheng.w@gmail.com; 5 Department of Mechanical Engineering, National Taipei University of Technology, Taipei 10608, Taiwan

**Keywords:** major depressive disorder, electroencephalography (EEG), brain-computer interface (BCI), common spatial pattern (CSP), machine learning

## Abstract

Major depressive disorder (MDD) has become a leading contributor to the global burden of disease; however, there are currently no reliable biological markers or physiological measurements for efficiently and effectively dissecting the heterogeneity of MDD. Here we propose a novel method based on scalp electroencephalography (EEG) signals and a robust spectral-spatial EEG feature extractor called kernel eigen-filter-bank common spatial pattern (KEFB-CSP). The KEFB-CSP first filters the multi-channel raw EEG signals into a set of frequency sub-bands covering the range from theta to gamma bands, then spatially transforms the EEG signals of each sub-band from the original sensor space to a new space where the new signals (i.e., CSPs) are optimal for the classification between MDD and healthy controls, and finally applies the kernel principal component analysis (kernel PCA) to transform the vector containing the CSPs from all frequency sub-bands to a lower-dimensional feature vector called KEFB-CSP. Twelve patients with MDD and twelve healthy controls participated in this study, and from each participant we collected 54 resting-state EEGs of 6 s length (5 min and 24 s in total). Our results show that the proposed KEFB-CSP outperforms other EEG features including the powers of EEG frequency bands, and fractal dimension, which had been widely applied in previous EEG-based depression detection studies. The results also reveal that the 8 electrodes from the temporal areas gave higher accuracies than other scalp areas. The KEFB-CSP was able to achieve an average EEG classification accuracy of 81.23% in single-trial analysis when only the 8-electrode EEGs of the temporal area and a support vector machine (SVM) classifier were used. We also designed a voting-based leave-one-participant-out procedure to test the participant-independent individual classification accuracy. The voting-based results show that the mean classification accuracy of about 80% can be achieved by the KEFP-CSP feature and the SVM classifier with only several trials, and this level of accuracy seems to become stable as more trials (i.e., <7 trials) are used. These findings therefore suggest that the proposed method has a great potential for developing an efficient (required only a few 6-s EEG signals from the 8 electrodes over the temporal) and effective (~80% classification accuracy) EEG-based brain-computer interface (BCI) system which may, in the future, help psychiatrists provide individualized and effective treatments for MDD patients.

## 1. Introduction

Major depressive disorder (MDD) is a prevalent mood disorder with the presentation of persistent sadness, feeling of worthlessness, restlessness, and loss of interest in daily activities [1], and patients may also suffer problems in decision making and concentration [2]. According to the World Health Organization (WHO), more than 350 million people suffer from major depression [3]. MDD has therefore become the leading contributor to the global burden of disease [4]. Despite the high impact on human health, the overall remission rates of treatments with either psychopharmacological or psychotherapeutic therapies for MDD are modest [5,6]. One possible reason for sub-optimal treatment response is the heterogeneous etiology of MDD, which is a phenomena based rather than a laboratory-test based diagnosis [1]. Biomarkers that include neuroimage, genetic fingerprint, proteomics, neuroendocrine function tests, and electrophysiological measurements, may help elucidate the endophenotypic signs for refining diagnosis and even unraveling the whole picture of MDD [7,8].

Among the candidate endophenotypic markers, neuroimaging evidence has suggested that there is a close association between MDD and abnormal structural changes of brains (e.g., reduced hippocampus volumes [9,10]). Abnormal brain activities were also observed in some subcortical regions of the limbic system (e.g., amygdala and thalamus [11]) and some cortical regions (e.g., the posterior lateral and medial orbitofrontal cortex regions [12]). Therefore, measuring brain activity is a reasonable approach to explore the endophenotype of MDD. Measurement of brain activity may help us refine MDD into several more homogeneous subgroups and to match an effective treatment [13,14]. The first step to find a valid endophenotypic marker for subgrouping patients with MDD is to have a biomarker which is valid to classify between patients with MDD and healthy controls.

Previously, there have been many EEG-based studies presented to investigate the difference in EEG between patients with MDD and controls or to classify the two groups with machine learning techniques [15,16,17,18,19,20,21,22,23,24,25,26,27]. Several earlier studies reported that there were significant spectral-spatial differences in electroencephalography (EEG) between patients with MDD and healthy controls [15,16,17,18]. With the rapid development of brain-computer interface (BCI) techniques, designing classification methods based on machine learning techniques has become a key research topic. Lee et al. [19] performed a detrended fluctuation analysis (DFA) on EEG signals from 11 patients with MDD and 11 healthy participants and observed a higher value of DFA feature in the MDD group. Hosseinifard et al. [20] tested the performance of classifying between patients with MDD and healthy controls with five different features extracted from each of the 19 EEG recording sites, including EEG spectral power, DFA, Higuchi fractal dimension, Grssberger and Procaccia (GP) algorithm-based fractal dimension (also called correlation dimension), and Lyapunov exponent. Their results showed that the GP fractal dimension (GPFD) could achieve a classification accuracy as high as 83.3% between patients and healthy controls, which outperformed the other four types of features. Li et al. [21] extracted 20 types of EEG features from each of the 128 channels and selected the best 20 features out of the 2560 (20 types ×128 channels) feature candidates. With the best 20 features, a *k*-nearest neighbor (*k*-NN) classifier-based classification accuracy of 99.1% was achieved with nine depressive and nine non-depressive participants (the depressive participants in [21] were not clinically diagnosed as patients, but labeled according to the BDI scores).

Although some previous works have proven the feasibility of the use of EEG in classifying between patients with MDD and healthy controls, several issues remain to be much improved. Two common limitations in the current literature are long acquisition time for valid EEG signals and long preparation time due to large numbers of recording sites. In order to increase the signal-to-noise ratio, the study of [20] recorded 5-min EEG from each subject and averaged the power spectrums of a set of 1-min EEG epochs segmented from the 5-min EEG signals. Band powers calculated from the averaged spectrum were the inputs into the classifier. One major drawback for a long EEG recording session would be that subjects tend to feel bored or even fall asleep. On the other hand, although gel-based systems have good data quality, the preparation (applying electric-gel to the electrodes, etc.) would be time consuming if there are too many recording sites. Therefore, how to achieve high classification accuracy with shorter recoding time and fewer EEG recording sites is a critical issue to address. In the present study, the inputs into the classification system are a few single-trial EEG signals of 6 s long. The aim of this study is to develop an effective feature extraction method capable of extracting discriminating features from the 6-s single-trial EEG signals with as few required recoding sites as possible by introducing the method of common spatial pattern (CSP).

Single-trial raw EEGs are known to have a poor spatial resolution due to the volume conduction effect [28]. To improve the signal-to-noise ratio of single-trial EEG, spatial filtering is necessary. Common spatial pattern (CSP) is a spatial filtering technique for single-trial EEG. It is based on the decomposition of the signal parameterized by a matrix that projects the signals in the original recording space to the ones in the surrogate sensor space where a set of transformed signals are optimal for discrimination between two classes [29,30], and has been proven to be better than other spatial filters such as bipolar and the common average reference filters [31]. Recently, CSP has gained wide acceptance in motor imagery BCI studies where the inputs were single-trial EEGs [32,33,34]. However, the classification performance based on CSP features highly depends on the operational EEG frequency bands of CSP. Therefore, selection of operational frequency bands for CSP is of primary importance.

Knott et al. [15] analyzed the EEG differences of asynchrony and asymmetry between depressed patients and controls. Their results revealed a reduced left-hemisphere alpha (8–13 Hz) power and widespread reduced theta (4–8 Hz), alpha, and beta (13–30 Hz) coherence measures in controls. In the study of Omel’chemko et al. [16], depressed patients versus controls demonstrated a significant increase in the relative power (i.e., anterior to posterior regions of the brain) of theta band, and a significant decrease in alpha and beta bands. Works by [18,19] also revealed similar power differences in theta, alpha and beta bands between patients with depressive disorders and healthy controls. Although the gamma band (30–44 Hz) was not included in the analysis of these studies [15,16,18,19], previous literature on EEG-based emotion recognition has indicated the gamma band to be an important feature in the classification between the high valence and low valence emotional processing [35]. Since the ability of emotion regulation for patients with depressive disorders seems to decline [11], it is reasonable to infer that the EEG gamma band may also be a crucial feature candidate. All these previous findings suggest that the oscillatory brain activities of theta, alpha, beta, and gamma bands may be good indicator for classifying between patients with depressive disorders and controls. However, it is unclear which bands or even sub-bands within the range of 4–44 Hz are the decisive keys to the depression-control classification based on CSP features. A strategy combining filter-bank CSP and transformation technique is adopted in this study in order to avoid exhaustive searching. Based on this strategy, we propose an EEG feature extraction method called kernel eigen-filter-bank CSP (KEFB-CSP).

The KEFB-CSP first extracts the CSP features from all possible sub-bands covering the range from theta, alpha, beta to gamma bands, and then transforms the filter-bank CSP features into a smaller set of nonlinear principal components by applying the kernel principal component analysis (KPCA) [36]. KPCA is a nonlinear extension of the regular PCA and performs PCA in a higher-dimensional feature space induced by a kernel. Different from the PCA which only computes the linear dependence between two variables, KPCA calculates higher-order statistics among variables [36,37], meaning that the KEFB-CSP is able to extract nonlinear dependencies among the filter-bank CSP features of multi-channel EEGs, which is crucial because EEG has the property of nonlinearity in pattern distribution [38]. Therefore, the proposed KEFB-CSP can carry informative spectral-spatial features of EEGs to achieve higher depression-control classification accuracy than filter-bank CSP and its linear transform by PCA.

In this study, we compare the proposed KEFB-CSP with other features, including EEG band powers (BP), band-specific CSP (i.e., the CSP of a one-single frequency band), and the GPFD which had been shown to be better than other features in [19]. Three kinds of commonly used classifiers are also compared, including *k*-NN, linear discriminant analysis (LDA), and support vector machine (SVM) [39] classifiers. Moreover, we also partition the electrode montage into several scalp regions where the numbers of the EEG recording sites in these regions are limited (between three and eight), and then compare the classification performances among these regions to find the best representative region with only a few EEG recorded sites for depression detection.

## 2. Materials and Methods

### 2.1. Participants

The whole experiment and EEG data acquisition were conducted in the National Taiwan University Hospital (NTUH), Taipei, Taiwan. Twenty-four subjects (12 patients of MDD and 12 healthy controls) participated in this study and they all provided signed informed consent approved by the Institution Review Board at NTUH (201203036RIB). The patient participants (four males and eight females) were aged from 43 to 70 years, with an average of 57 (SD=7.71). Controls were age- and sex-matched. All patients were diagnosed by a board certified psychiatrist based on the diagnostic criteria of major depressive disorder according to the Diagnostic and Statistical Manual of Mental Disorders Fourth Edition Text Revision (DSM-IV-TR). For each patient, diagnosis of MDD was confirmed, and psychiatric comorbidities were evaluated by using the Mini International Neuropsychiatric Interview (MINI) [40].

### 2.2. Apparatus, Settings, and Preprocessing

All emotional stimuli and experimental cues were presented on a 17-inch CRT screen. Participants were seated in a fixed and comfortable chair. The distance between the eyes of each participant and the CRT display were controlled to be about 60 cm. EEG signals were recorded by 33 Ag/AgCl electrodes mounted on an electro-cap (Quick-Cap 32, NeuroScan Inc., Charlotte, NC, USA) where the locations of the electrodes follow the international 10–20 system. Among the 33 channels, two were references positioned at A1 (left mastoid) and A2 (right mastoid), and one is a Ground channel at forehead, leaving a total of 30 EEG channels used in this study. Impedances of the electrodes were kept below 5 kΩ by applying Quick-Gel (Compumedics Inc., Charlotte, NC, USA). Ocular artifacts were monitored by horizontal (HEOR, HEOL) and vertical (VEOH, VEOL) bipolar EOG electrodes. We used artifact removal software of NeuroScan to remove eye blinks and eye-movements related artifacts embedded in the recorded EEG. The artifact removal is based on an EEG-VEOG covariance analysis, a linear regression procedure, and the creation and use of an LDR file to perform a point-by-point proportional subtraction of the eye blinks [41]. Participants were asked to maintain a minimum muscle activity in order to prevent possible artifacts caused by electromyography. The recorded EEG and EOG were filtered on-line with a band pass filter (0.5–100 Hz) and sampled at 500 Hz using the NuAmps amplifier from NeuroScan.

### 2.3. EEG Data Collection

In the current study, we collected EEGs from a resting state as the feature basis for classification. Each participant underwent two sessions of EEG acquisition with each session contained three trials of the resting state. Prior to each trial of resting state, there was a 5-s ready-to-start period during which the participants could make very slightly body/eye movements. For each trial (lasted for 54 s) in the resting state, participants were asked to relax and loosely look at the central fixation cross shown in the monitor screen while not to think anything purposefully. They were also asked to maintain a minimum arousal level without falling into sleep. The EEG recorded in each trial of resting state was further partitioned into nine resting EEG epochs of 6-s length. There was a break between two sessions. The length of the break period was 5–10 min, depending on the participants. As a result, for each participant, there were 54 EEG signals of 6 s from the two EEG acquisition sessions.

### 2.4. Feature Extraction

In this subsection, we first introduce the features to be compared, including BP and GPFD, and then derive the proposed KEFB-CSP method in detail. Major depressive disorder group and the healthy control group are defined as positive class (class 1) and negative class (class 2), respectively. Vectors and matrices are marked as boldface.

#### 2.4.1. BP

The spectrum of a 6-s EEG signal from one channel were calculated by discrete Fourier transform and powers of theta (4–8 Hz), alpha (8–13 Hz), beta (13–30 Hz), and gamma (30–44 Hz) bands were extracted. In the experimental results, for example, the theta band power (theta BP) feature is an *N*-dimensional vector in which the elements are the theta BPs computed from the EEGs of *N* different channels.

#### 2.4.2. GPFD

Fractal dimension of a time series can be measured by GP algorithm. Let x=[x(1), x(2), …, x(T)] be a time series of length *T*, where T=6s ×500 Hz=3000  sample points in this study. The time series can be reconstructed as a set of *m*-dimensional vectors as:

(1) y(i)=[x(i),x(i+τ),x(i+2τ),…,x(i+(M−1)τ)], i=1,2,…, T−(M−1)τ

where τ is the time delay and m is the embedding dimension. The correlation integral is given by:

(2) $$C\left(r\right)=\frac{2}{T\left(T-1\right)}\overset{T-\left(M-1\right)\tau }{\underset{\begin{array}{c} i,j=1\\ i\neq j \end{array}}{\sum }}\theta \left(r-\left|y\left(i\right)-y\left(j\right)\right|\right)$$

where θ is a Heaviside step function defined as θ(y)=1 if y>0; θ(y)=0 otherwise. The value of the correlation integral represents the probability that the set of |y(i)−y(j)| falls into the cell of size r at a given value of the embedding dimension M. The correlation dimension is given by:

(3) $$d_{c}\left(M\right)=\underset{r\to 0}{\lim}\frac{\log C\left(r\right)}{\log r}$$

In numerical computation, the value of $d_{c}$ is obtained by estimating the slope of logC(r) versus logr. However, too small values of r result in zero C(r) values, and too large values of r would lead to C(r) values which are all equal to a constant. Therefore, given a value of M, the slope (i.e., $d_{c}$) should be estimated over the region where logC(r) is approximately linear in logr by linear least-squares fitting, as suggested in [20]. Further, the value of $d_{c}\;$ gradually increases as the value of M increases, and then achieves saturation. The saturation value of $d_{c}$ is the GPFD of the time series x. In the current study, an EEG signal had 3000 sample points (T=6 s×500 Hz=3000). According to our preliminary tests, the $d_{c}$ values achieved saturation when the value of M was 20 ~ 30 for all signals. Therefore, the value of M varied from 2 to 30 in searching the GPFD in this study. On the other hand, the parameter τ satisfies the condition: T−(M−1)τ>0 according to Equation (1). Since the maximum value of M was set as 30, the maximum τ value was 102. The best time delay τ needs to be determined experimentally, by cross validation for example. For N-channel EEG signals of a trial, N GPFD features value were extracted, and these GPFD values are concatenated to an N-dimensional vector.

#### 2.4.3. KEFB-CSP

**Figure 1:**
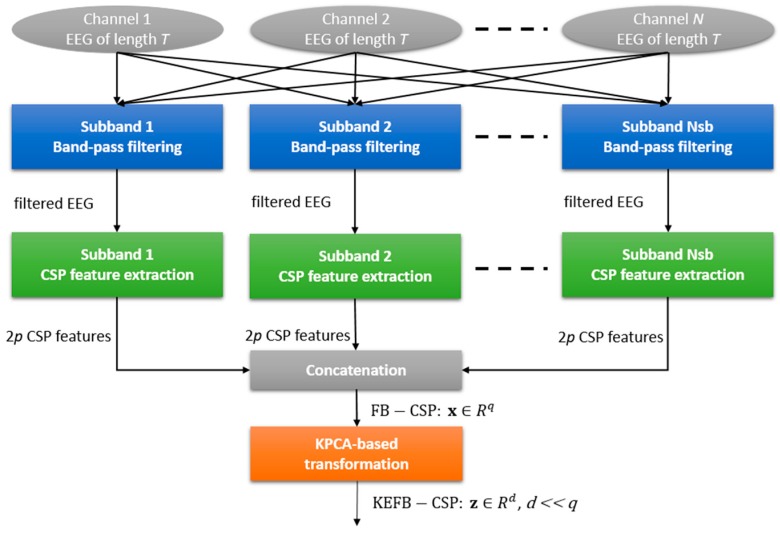
Illustration of the EEG feature extraction method of kernel eigen-filter-bank common spatial pattern (KEFB-CSP).

The KEFB-CSP method is composed of three stages, including band-pass filtering, filter-bank CSP extraction, and KPCA-based transformation, as illustrated in Figure 1.

Stage 1: Band-pass Filtering

The frequency band ranging from theta to gamma bands (4–44 Hz) is partitioned into $N_{sb}$ sub-bands with equal width (2 Hz or 4 Hz width). If the sub-band width is 4 Hz, then $N_{sb}=10$, and the 10 subbands includes 4–8 Hz, 8–12 Hz, 12–16 Hz, 16–20 Hz, 20–24 Hz, 24–28 Hz, 28–32 Hz, 32–36 Hz, 36–40 Hz, and 40–44 Hz. On the other hand, $N_{sb}=20$ if the width of each sub-band is 2 Hz. A 6-s single-trial multi-channel EEG is band-pass filtered to generate EEGs of the 10 indicated sub-bands using a set of 3rd-order Butterworth filters. The filtered EEGs are sent into the second stage for filter-bank CSP feature extraction.

Stage 2: Filter-bank CSP Feature Extraction

Given a filtered N-channel EEG E  (represented by an N×T matrix), the method of CSP aims to find a matrix W to transform the filtered EEG E  in the original sensor space into new time series $E_{N}$ in a surrogate sensor space by the following five steps:

*Step 1*. Calculate the averaged spatial covariance matrices of the two classes ${\bar{C}}_{1}$ and ${\bar{C}}_{2}$:
  (4) $${\bar{C}}_{1}=\frac{1}{n_{1}}\overset{n_{1}}{\underset{i=1}{\sum }}\frac{E_{1i}E_{1i}^{t}}{tr\left(E_{1i}E_{1i}^{t}\right)}\;,\;{\bar{C}}_{2}=\frac{1}{n_{2}}\overset{n_{2}}{\underset{i=1}{\sum }}\frac{E_{2i}E_{2i}^{t}}{tr\left(E_{2i}E_{2i}^{t}\right)}$$
  where *t* denotes the transpose of a matrix, *tr*(.) denotes the trace of (.), $E_{1i}$ is the *i*th training EEG data of class 1, $E_{2i}\;$ is the *i*th training EEG of class 2, and $n_{1}$ and $n_{2}$ are the numbers of the training EEG data of the first and second classes, respectively. Notice here that an EEG training data is a N×T signal matrix.
*Step 2*. Diagonalize the composite covariance $\bar{C}$ by $\bar{C}=U\lambda U^{t}$, where $\bar{C}={\bar{C}}_{1}+{\bar{C}}_{2}$, U is the matrix in which the columns are the orthonormal eigenvectors of $\bar{C}$, and λ is an N×N diagonal matrix in which the diagonal elements are the eigenvalues sorted in descending order.
*Step 3*. Whiten the averaged spatial covariance metrics of the two classes by $S_{i}=P{\bar{C}}_{i}P^{t}$, *I* = 1,2, where $P=\lambda^{-1/2}U^{t}$ is the whitening matrix.
*Step 4*. Perform the simultaneous diagonalization of the whitened spatial covariance matrices:
  (5) $$S_{1}=B\lambda_{1}B^{t},S_{2}=B\lambda_{2}B^{t}$$
  where $S_{1}$ and $S_{2}$ share the same eigenvectors, and $\lambda_{1}+\lambda_{2}$ equals an identity matrix I, indicating that the eigenvectors having larger eigenvalues for $S_{1}\;$ have smaller ones for $S_{2}$.
*Step 5*. Projecting the whitened EEG of a trial onto the eigenvectors in B yields new *N*-channel signals $E_{N}$ (an N×T matrix):
  (6) $$E_{N}=B^{t}\left(PE\right)=WE$$

Thus, the CSP transformation matrix is given by $W=B^{t}P$.

The first *p* and the last *p* rows of $E_{N}$ are new signals which are optimal for the classification of the two classes. The CSP features are the normalized log powers of the 2*p* new signals $z_{i}$:

(7) $$csp_{i}=\log\left(\frac{var\left(z_{i}\right)}{\sum_{i=1}^{2p}var\left(z_{i}\right)}\right),i=1,\ldots ,2p$$

where var denotes the squared sum of the amplitudes of the new signal $z_{i}$.

For each frequency sub-band, 2*p* CSP features are extracted from a filtered multi-channel EEG as a result. The CSP features extracted from the $N_{sb}$ sub-bands are concatenated to a feature vector of dimension q, where $q=2p\times N_{sb}$. This vector is a filter-back CSP (FBCSP). Moreover, the number of chosen new signals 2*p* needs to be determined experimentally, and the optimum value of *p* should be searched within the range from 1 to N/2 (if the number of channels N is even) or (N−1)/2 (if N is an odd value).

Stage 3: KPCA-based Transformation

Let $S={\left\{x_{i}\right\}}_{i=1,\ldots ,n}$ be the training set containing the FB-CSP data $x_{i}\in R^{q}$ from both classes, where $n=n_{1}+n_{2}$ is the size of the set. KPCA maps the data into a higher-dimensional feature space *F* using a nonlinear mapping φ, and then performs PCA in this mapped space. KPCA diagonalizes the covariance of the centered data in the space *F*:

(8) $$\lambda v=\Lambda v=\left(\frac{1}{n}\overset{n}{\underset{i=1}{\sum }}\varphi \left(x_{i}\right)\varphi {\left(x_{i}\right)}^{t}\right)v$$

where Λ is the covariance of the mapped training data, v is the eigenvector associated with eigenvalue λ, and is spanned by the mapped training set:

(9) $$v=\overset{n}{\underset{i=1}{\sum }}a_{i}\varphi \left(x_{i}\right)$$

where $a_{i}$ are the coefficients of the expansion. Notice that the mapped data $\varphi \left(x_{i}\right)$ in Equation (9) ave zero mean in space *F*, i.e., $\sum_{i=1}^{n}\varphi \left(x_{i}\right)=0$. Solving the eigenvalue problem in Equation (8) is equivalent to solving the eigenvalue problem as:

(10) $$n\lambda a=\bar{K}a$$

where $a={\left[a_{1},a_{2},\ldots ,\;a_{n}\right]}^{t}$ is the eigenvector in which the elements are the expansion coefficients in Equation (9), and the n×n centered kernel matrix $\bar{K}$ is given by:

(11) $$\bar{K}=K-1_{n}K-K1_{n}+\;1_{n}K1_{n}$$

in which the matrix $1_{n}={\left(1/n\right)}_{n\times n}$ containing entries which are all equaling to one, and $K=\left(K\left(x_{i},x_{j}\right)\right)$ is the n×n kernel matrix with entries $K\left(x_{i},x_{j}\right)$ defined by the inner product of two mapped data. In this study we chose the Gaussian function as the kernel for KPCA:

(12) $$K\left(x_{i},x_{j}\right)=e^{-\frac{\|x_{i}-x_{j}\|{}^{2}}{2\sigma^{2}}}$$

where σ is the width of the kernel function. Calculate the orthonormal eigenvectors $a_{1},a_{2},\ldots ,\;a_{d}$ of $\bar{K}$ corresponding to the largest positive eigenvalues $\lambda_{1}\geq \lambda_{2}\geq \ldots \;\geq \lambda_{d}$, where $a_{i}$ are normalized by $a_{i}=a_{i}/\sqrt{\lambda_{i}}$, and 1≤d≤n. The *d* leading eigenvectors of the covariance Λ corresponding to the eigenvalues $\lambda_{1},\lambda_{2},\ldots ,\lambda_{d}$ are thus given by:

(13) $$v_{i}=Qa_{i},i=1,\ldots ,\;d$$

where $Q=\left[\varphi \left(x_{1}\right),\;\varphi \left(x_{2}\right),\ldots ,\varphi \left(x_{n}\right)\right]$.

For a test data x, its projections onto the *d* eigenvectors of the covariance are computed by:

(14) $$z_{i}=v_{i}^{t}\varphi \left(x\right)\;=a_{i}^{t}Q^{t}\varphi \left(x\right)\;=a_{i}^{t}{\left[K\left(x_{1},x\right),K\left(x_{2},x\right),\ldots ,\;K\left(x_{n},x\right)\right]}^{t},i=1,\ldots ,\;d$$

The projections $z_{i},\;i=1,\ldots ,\;d$ are nonlinear principal components of the filter-bank CSP features and form a *d*-dimensional feature vector z called a KEFB-CSP.

### 2.5. Parameter Optimization and Participant-Independent Classification

In order to obtain the participant-independent classification accuracy, the leave-one-participant-out cross validation (LOPO-CV) procedure described in Table 1 was performed in the experiments.

In step 3, a *k*-NN classifier was employed to compute the classification rate, where the number of nearest neighbors *k* was set as 3. The parameters of the feature extractions methods are summarized in Table 2, where the values to be searched for each parameter are also listed. In Table 2, the CSP refers to the CSP feature extracted from one single-band (theta, alpha, etc.) EEG. The parameters of each method were optimized using the grid search method combined with the LOPO-CV procedure. The parameter space of the method was partitioned into girds, and each grid was a combination of the parameters, $\left(d,\sigma \right)=(1,2^{-3}$) for example. For each cell of the grids, the LOPO-CV procedure was performed to compute the corresponding CV accuracy. The optimum value combination of the parameters that resulted in the highest CV accuracy were selected. For example, the optimum value of *d* represents the minimum number KEFB-CSP features. The accuracies reported in the following results were the best CV accuracies.

## 3. Results and Discussion

### 3.1. Comparison with Existing EEG Features for MDD Detection

**Figure 2:**
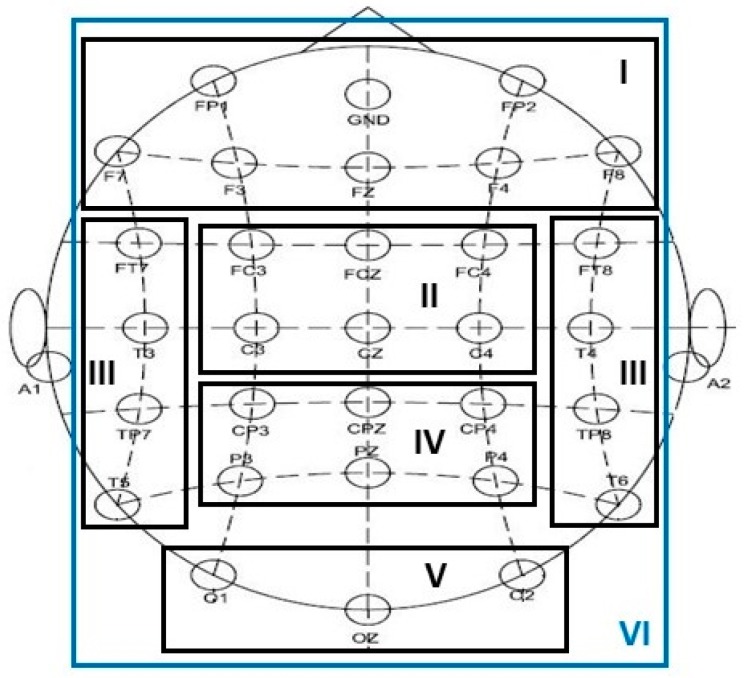
Six different montages selected for comparison in this study. The montages from **I** to **VI** cover the frontal, central, temporal, parietal, occipital, and entire scalp areas of the brain.

We selected six electrode montages covering different scalp regions in order to compare the differences between patients with MDD and controls (see Figure 2). The montages from I to VI cover the frontal, central, temporal, parietal, occipital, and entire scalp areas of the brain. For each montage, the number of channels *N* is listed in Table 3. Previously, the BP, coherence, and GPFD have been used as the features for classifying the two groups. Coherence is a measure for the synchrony between EEG activities at different locations. It is a function of frequency for two EEG signals from different channels, and has the value within the interval between 0 and 1. For a frequency band/point and an *N*-channel EEG configuration, a total of (N×N−N)/2  coherence features can be obtained, and these features form a coherence feature vector. In the following, we compare the proposed KEFB-CSP feature extraction method with these three features as well as the conventional CSP and FBCSP features. The comparison of classification accuracies among the feature extraction methods at different scalp areas are listed in Table 4, where the accuracies were obtained by *k*-NN classifier. Please note that the feature dimensions of the six areas are different. Taking the theta BP as an example, the feature dimension is 30 if the area of interest is the “entire” (i.e., montage VI) but only 7 if the region is the “frontal” (i.e., montage I).

According to Table 4, the best accuracies achieved by the delta (0.5–4 Hz), theta, alpha, beta, and gamma BPs are 61.03%, 59.02%, 68.98%, 59.49%, and 60.33%, respectively. Among the different bands, alpha performs the best in terms of BP feature. Both coherence and GPFD show no discriminating ability in classifying the two groups. The accuracies of coherence and GPFD in all scalp regions are close to the chance level of 50%. As for CSP, the best CSP accuracies for the delta, theta, alpha, beta, and gamma bands are 57.64%, 59.56%, 64.58%, 69.98%, and 64.66%, respectively. Overall, the CSP method outperforms both coherence and GPFD, and is slightly better than BP for all bands except for alpha band. We considered two types of FBCSPs: sub-bands of 4 Hz width (partitioning 4–44 Hz into 10 sub-bands) and the ones of 2 Hz width (20 sub-bands in total). The best 4-Hz and 2-Hz FBCSP accuracies are 70.45% and 69.44%, respectively, and were obtained from the temporal area. Among the five scalp regions, 4-Hz FBCSP performs better than 2-Hz FBCSP in three regions including central, temporal, and parietal regions. Nevertheless, the differences in accuracies between the two types of FBCSP were small. As far as the computational complexity is concerned, the 4-Hz FBCSP is preferred, because 4-Hz FBCSP only needs to perform the CSP computation for 10 times (for 10 sub-bands) while 2-Hz FBCSP needs to perform the CSP for 20 times. The method of 4-Hz FBCSP is therefore much more computationally cheaper. On the other hand, the 4-Hz FBCSP only outperformed the best CSP (i.e., beta CSP) by 0.47% (70.45−69.98%). However, the former required only EEG data from 8 channels located at the temporal region while the latter required data from 30 channels of the entire scalp area. Practically, 4-Hz FBCSP is thus more feasible because the use of 8 channels can result in much shorter system setup time. Therefore, we only considered the 4-Hz case in the FBCSP+PCA and the proposed KEFB-CSP in our experiment. Also, the 4-Hz FBCSP will be simply referred to as FBCSP hereafter.

Next, we compare the accuracy among FBCSP, FBCSP+PCA, and KEFB-CSP. Here, the PCA is implemented by replacing the kernel PCA’s Gaussian kernel with the polynomial kernel $K\left(x_{i},x_{j}\right)={\left(x_{i}\cdot x_{j}\right)}^{c}$, and then setting the degree of the polynomial to 1 (setting c=1). By doing so, kernel PCA is identical to PCA because the feature space is identical to the original input space (i.e., no mapping). In other words, FBCSP+PCA is in fact a special case of the proposed KEFB-CSP. By applying the PCA to reduce the dimension of FBCSP (i.e., FBCSP+PCA), the accuracy is improved for all scalp regions. The best result of 75% for FBCSP+PCA was obtained from the temporal area, and was much better than that for FBCSP in the same scalp region (70.45%). Finally, the accuracy was further improved for all scalp regions by replacing PCA with kernel PCA (i.e., KEFB-CSP), as can be seen in Table 4. The best accuracy of KEFB-CSP was 77.08% (also at temporal region). The above results show that the proposed KEFB-CSP feature is superior to the existing features (BP, coherence, and GPFD) that had been previously applied to the EEG classification between depressed patients and controls, and also performs better than the conventional band-specific CSP and FBCSP in terms of classification accuracy of single-trial EEG.

### 3.2. Comparison with Different Classifiers in Single-Trial Analysis

**Figure 3:**
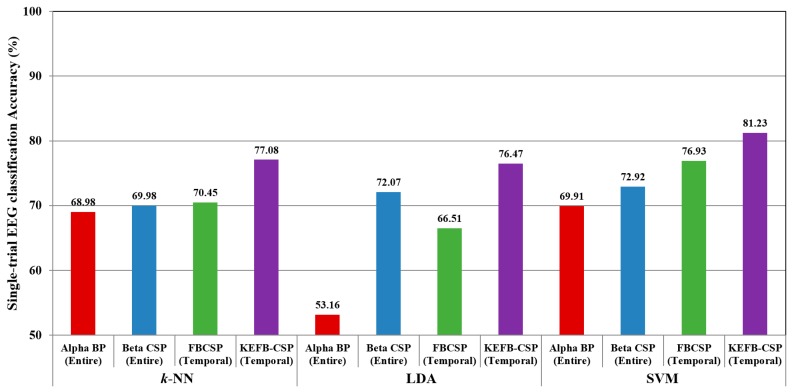
Comparison of average classification accuracies over 54-trial EEGs among different classifiers.

In this subsection, we further compare the *k*-NN classification results with the results by LDA and SVM classifiers. LDA involves no free parameters [38]. SVM has two parameters: the penalty weight and the parameter of the kernel function [34,38]. In this study, the Gaussian function in Equation (12) was also adopted as the kernel for SVM. The parameters of SVM were all optimized using the LOPO-CV procedure combined with grid searching method. During the optimization, the optimum value of the SVM penalty weight (denoted as *C*) was found in the set {10, 50, 100}, and the optimum value of the SVM kernel parameter (denoted as $\sigma_{s}$) were searched within the range from 0.01 to 100. In this comparison, only the best condition (i.e., band + scalp region or just scalp region) for each feature was considered. According to Table 4, the best conditions for BP, CSP, FBCSP, and KEFB-CSP are “alpha+entire”, “beta+entire”, “temporal”, and “temporal”, respectively. The results are shown in Figure 3.

Figure 3 shows that the LDA was not necessarily better than *k*-NN, depending on the use of feature. Compared with *k*-NN, LDA achieved better accuracy (72.07%) for beta CSP feature; however, LDA gave a worse accuracy (53.16%) for the alpha BP feature. One possible reason is that the distributions of the two groups are nonlinearly distributed in the space of alpha BP patterns, making the LDA unable to effectively classify the two groups because LDA is essentially a linear classifier. And therefore, we did not consider the LDA classifier in the subsequent experiments. By contrast, the nonlinear classifier SVM achieved better results for all features. Also, KEFB-CSP outperforms the other three (alpha BP, beta CSP, FBCSP) for all classifiers. According to the results in Figure 3, the highest classification accuracy 81.23% was achieved by the combination of KEFB-CSP feature and the SVM classifier.

### 3.3. Individual Classification Using Majority Voting Strategy-Based LOPO-CV

The results reported in Section 3.1 and Section 3.2 are all based on single-trial analysis. The results provide clear evidence that the proposed KEFB-CSP feature outperforms other features in single-trial analysis. In this subsection, we further discuss the following two questions:
*Question 1*:How many participants can be correctly classified based on the use of KEFB-CSP feature?*Question 2*:Can the individual classification accuracy be improved by using multiple single-trial EEG signals?

To answer these two questions, we designed a voting-based LOPO-CV procedure which can estimate the participant-independent individual classification accuracy. The steps of this procedure are summarized in Table 5. The voting strategy performed in Step 3 is based on the criterion that a testing participant is correctly classified if more than 50% of the EEG signals from his/her *first* $n_{t}$ trials are correctly classified.

The LOPO-CV (Table 1) estimates the average classification accuracy of single-trial EEGs (i.e., an EEG epoch of 6 s), whereas the voting-based LOPO-CV (Table 5) estimates the accuracy representing the probability that one individual is correctly classified. Please also note that for each case of $n_{t}$, the optimum parameters of the method should be found independently by using grid searching method, based on the criterion that the optimum parameters should resulted in the highest voting-based LOPO-CV accuracy (i.e., individual classification accuracy).

Table 6 and Table 7 show the experimental results of the voting-based LOPO-CV in $n_{t}=15$ and $n_{t}=1$, respectively. In the two tables, participants 1–12 were patients with major depressive disorder (denoted as D), and participants 13–24 were healthy controls (denoted as H). As $n_{t}=15$ (Table 6), 10 participants were classified correctly in the patient group, and thus the sensitivity was 83.33%. Similarly, 10 participants were classified correctly in the control group, and thus the specificity was 83.33%. Overall, the individual classification accuracy was 83.33% as $n_{t}=15$. On the other hand, as $n_{t}=1$ (Table 7), the sensitivity achieved 100% and the individual classification accuracy was higher than 90% (91.67%), with only two participants (both were healthy controls) were misclassified. By further comparing the results of the two tables, it can be found that the 15 EEG signals of participant 4 were all misclassified in the case of $n_{t}=15$ (Table 6); however, the patient’s first trial’s EEG was correctly classified in the case of $n_{t}=1$ (Table 7). This was due to the fact that the kernel methods’ (SVM for example) performance is very sensitive to the value of the kernel parameters. Choosing different values for the kernel parameters is equivalent to mapping the same data set into different feature spaces [39], meaning that the SVM decision boundaries in different settings of the kernel parameters would be different. As shown in Table 8, the optimum values of the SVM kernel parameters are different in the two cases of $n_{t}=15$ and $n_{t}=1$. Therefore, some misclassified participants in the case of $n_{t}=15$ could be correctly classified ones in the case of $n_{t}=1$.

**Figure 4:**
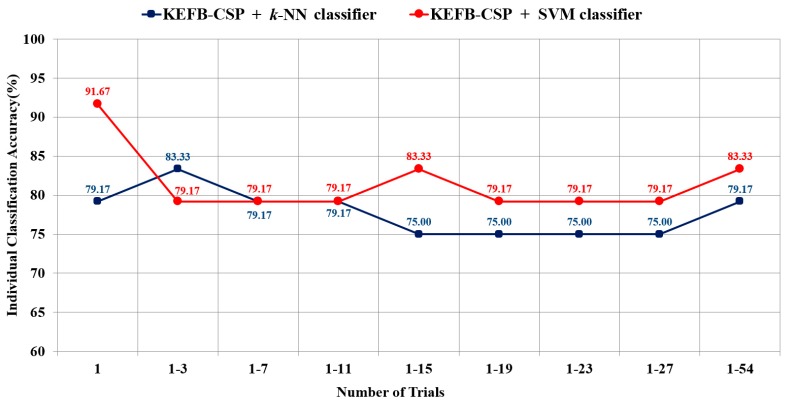
Voting-based individual classification accuracy versus the number of the first $n_{t}$ trials. The KEFB-CSP features were all extracted from the eight electrodes over the temporal scalp area.

Based on the voting-based LOPO-CV, we conducted an experiment in which the number of the first $n_{t}$ trials varied from 3 to 27 with a step size of 4. The extreme cases of $n_{t}=1$ and $\;n_{t}=54$ were also involved in this experiment. As $n_{t}=1$, the ratio calculated in Step 3 would be either 0 or 1 because there was only one 1 EEG signal for each participant. The experimental results of the individual classification accuracy versus different $n_{t}$ numbers are illustrated in Figure 4. The optimum parameters for the KEFP-CSP and SVM in each case are listed in Table 8. In Figure 4, the accuracies of 91.67%, 83.33%, and 79.17% represent that 2, 4, and 5 out of the 24 participants were misclassified, respectively. As shown in Figure 4, SVM outperforms *k*-NN is most cases. The results also reveal that both SVM and *k*-NN classifiers achieved their best performance with small numbers of $n_{t}$: *k*-NN achieved the highest accuracy 83.33% when $\;n_{t}=3$, and SVM achieved the highest accuracy 91.67% when $n_{t}=1$. In summary, a mean individual classification accuracy of about 80% can be achieved by using the proposed KEBF-CSP feature, SVM classifier and less than 7 trials, as shown in Figure 4. Such results suggest that it is possible to achieve reasonably high classification accuracy with a short period of EEG recording time in clinical practices.

### 3.4. Discussion

In our study, we have proposed a novel EEG feature called kernel eigen-filter-bank common spatial pattern (KEFB-CSP), and compared the KEFB-CSP with other features that have been applied in previous MDD studies, including the BPs and the fractal dimension features. Moreover, previous studies about the discrimination between MDD patients and normal subjects did not conduct experiments to analyze the performance difference between electrodes of different scalp areas in the two-class classification (MDD vs. normal) based on machine learning methods. In our study, we conducted such experiment and found that the temporal area is able to achieve higher accuracy than other areas. The advantage of this analysis is two-fold. First, the fact that only eight channels provides the highest classification accuracy suggest a BCI system with low numbers of electrodes is feasible for classification of MDD vs. healthy controls. Second, this finding (temporal area is more sensitive) also points to potential directions for neuroscientists to further investigate the difference in brain activities between MDD patients and normal individuals.

The proposed KEFB-CSP method has several advantages. First, it is able to achieve higher classification accuracies than the existing feature extraction methods for classification between MDD patients and normal subjects. Second, it requires only eight channels over the temporal area to achieve the highest accuracy (81.23%). Compared with BP which requires 30 channels covering the entire brain area to achieve its optimum performance (69.91%), the proposed KEFB-CSP requires a much smaller number of EEG channels. However, the proposed KEFB-CSP has a much higher computational complexity. In FB-CSP, the eigenvalue problem formulated in Equation (5) has the time complexity of $O\left(N^{3}\right)$, which needs to be solved for each sub-band. Since there are 10 subbands (4-Hz width each), this eigenvalue problem needs to be solved for 10 times. Also, in the stage of KPCA-based transformation (Stage 3), the eigenvalue problem described in Equation (10) needs to be solved again, which has the time complexity of $O\left(n^{3}\right)$. The drawback of this time-consuming computation further magnifies when the LOPO-CV procedure combined with the grid search method is performed to find the optimal values of the KEFB-CSP parameters. As a result, the training of the KEFB-CSP is extremely time consuming, which is the disadvantage of the propose method. This drawback could be dealt with by using parallel computing technique to speed up the training, which would be particularly needed when our method is tested with more subjects.

In Taiwan, the sensitivity and specificity of PHQ-9 for identifying MDD has been reported to be 86.0% and 91.1%, respectively [42]. The KEFB-CSP method has comparable performance to differentiate MDD case from controls, but its clinical utility is different from the self-reported measures. We preliminarily demonstrated the discriminant validity of this EEG-related biomarker for MDD. In the future, more studies which focus on the temporal stability and predictive validity of the KEFB-CSP method would help us to determine whether it can serve as a candidate endophenotypic sign to dissect the etiological heterogeneity and to provide MDD patients with individualized and effective treatment.

## 4. Conclusions

In the current study, we presented a novel EEG feature extraction method called kernel eigen-filter-bank common spatial pattern (KEFB-CSP) for the classification between patients with MDD and heathy controls. Based on the experimental results, several conclusions can be drawn. First, the spectral-spatial feature extraction method KEFB-CSP outperforms the conventional CSP, and other EEG features that had been used in related works, including the powers of different EEG frequency bands and the fractal dimension extracted from EEG time series. Second, KEFB-CSP feature is able to achieve a participant-independent individual classification accuracy of 91.67% when a nonlinear support vector machine (SVM) serves as the classifier, meaning that the probability that one person is correctly classified into the depression or control groups is higher than 90%. Lastly, the accuracy of 91.67% was achieved with only the first single-trial resting-state EEG signal of 6 s from 8 EEG electrodes located at the temporal area.

The successful implementation of the proposed method has several implications for future development of an EEG-based BCI system for major depression detection. First, the proposed method only requires eight electrodes to record EEG. According to our experience, the preparation time for an 8-electrode setting (applying electric-gel to reduce the electrode impedance, introducing the test procedures to users, etc.) can be shorter than 10 min. Users will not feel uncomfortable due to the short duration of electro-cap wearing. Second, the method only requires a few 6-s single-trial resting-state EEG signals for testing. Users do not need to experience a long and exhausted EEG data collection procedure. The two technical strengths increase the feasibility of its clinical application.

Moreover, the search for a suitable biomarker that can help elucidate the endophenotypic signs for refining the spectrum of MDD is therefore important for optimizing treatment plans. As a simple and efficient method to measure the brain electrophysiological activity, the proposed KEFB-CSP based EEG feature seems to have a potential to provide psychiatrists with a valid and reliable candidate biomarker that can assist psychiatrists to dissect heterogeneous etiology of MDD and to selectively adjust effective treatment plans optimized for different subgroups of MDD patients.

Nevertheless, several follow-up studies will be needed. First, only 24 subjects participated in the current study. To further validate the validity of the proposed method, more participants are needed in the future. Second, although the current results suggest that the temporal area is better than other scalp areas in the classification between MDD patients and healthy controls, a combination of EEG electrodes from multiple scalp areas might achieve even higher classification accuracy. For example, the combination of some electrodes over the temporal area and some electrodes over the parietal area may lead to a better classification performance. Future developments of an electrode selection algorithm based on machine learning technologies (e.g., Fisher’s separability criterion, mutual information, etc.) could provide an effective approach to find the optimum electrode combination across scalp areas. Finally, the mental state in which EEGs are recorded could be optimized. In the current study, the EEGs were recorded in the resting state. However, EEGs recorded in a different state may result in better performance for classifying between MDD patients and healthy controls. For example, it is evidenced that the emotion regulation ability for MDD patients seems to decline. Accordingly, the EEG signals recorded in the emotion-induction state may lead to better classification accuracy than the resting-state EEGs.

## Figures and Tables

**Table 1:** The leave-one-participant-out cross validation (LOPO-CV) procedure.

Initialize Free Parameter(s) of the Chosen Method
*Step 1*: 54 EEG data of the *i*th participant were used as the test data, and the 1242 (54×23) EEG data of the remaining 23 participants were all used for training the method. *Step 2*: Fed the 54 test data into the trained method and calculated the classification rate $R_{i}$. *Step 3*: Repeated step 1 and 2 until every participant was chosen as the testing participant once. *Step 4*: Calculated the average classification rate (i.e., CV accuracy) by $R_{ave}=(1/24)\times \sum_{i=1}^{24}R_{i}$.

**Table 2:** Parameters of the feature extraction methods.

Method	Descriptions	Values to Be Searched
BP	non	none
Coherence	non	none
GPFD	τ: time delay	τ={50,60,70, 80, 90, 100}
CSP	2p: number of chosen new signals	p={1,…, N/2} if N is even; p={1,…, (N−1)/2} if N is odd
FBCSP	2p: number of chosen new signals	p={1,…, N/2} if N is even; p={1,…, (N−1)/2} if N is odd
KEFB-CSP	2p: number of chosen new signals	p={1,…, N/2} if N is even; p={1,…, (N−1)/2} if N is odd
	d: number of chosen eigenvectors σ: width of the Gaussian kernel of KPCA	d={1,2,…, n}; σ is searched within the range from 1 to 100.

**Table 3:** Six electrode montages and the numbers of channels of each.

Brian Area (Montage)	Frontal (I)	Central (II)	Temporal (III)	Parietal (IV)	Occipital (V)	Entire (VI)
Channels	FP1, FP2, Fz, F3, F4, F7, F8	FCz, FC3, Cz, FC4, C3, C4	FT7, T3, TP7, T5, FT8, T4, TP8, T6	CP3, CPz, CP4, P3, Pz, P4	O1, Oz, O2	all channels
*N*	7	6	8	6	3	30

**Table 4:** Comparison of average classification accuracies over 54 trials of EEG signals among different features and scalp regions using *k*-NN classifier (in %).

EEG Features		Frontal	Central	Temporal	Parietal	Occipital	Entire
BP	Delta	61.03	50.23	55.32	57.02	48.15	55.02
	Theta	58.71	49.61	59.02	52.77	49.76	57.02
	Alpha	52.93	52.31	65.81	45.44	49.76	68.98
	Beta	48.68	53.08	59.49	54.55	41.97	50.30
	Gamma	43.59	43.67	60.33	54.24	52.16	44.29
Coherence	Delta	46.22	50.93	43.83	46.84	44.06	48.69
	Theta	47.22	49.69	42.67	48.07	46.76	51.93
	Alpha	52.01	51.39	47.45	46.53	49.07	57.10
	Beta	49.54	48.77	44.83	48.38	46.14	47.15
	Gamma	48.77	46.91	54.24	47.76	55.56	47.69
GPFD		49.53	43.13	44.83	42.59	54.55	36.57
CSP	Delta	49.31	52.16	55.02	57.64	47.22	53.01
	Theta	55.63	56.17	56.32	57.33	41.74	59.56
	Alpha	58.33	63.11	60.26	60.33	52.62	64.58
	Beta	47.99	59.95	60.80	56.40	50.69	69.98
	Gamma	50.23	43.05	64.66	61.57	53.62	64.66
FBCSP	4-Hz width	56.71	60.65	70.45	64.43	50.08	65.35
	2-Hz width	60.65	60.19	69.44	60.42	52.31	67.67
FBCSP+PCA	4-Hz width	60.11	66.67	75.00	65.28	58.18	69.75
KEFB-CSP	4-Hz width	62.73	69.29	77.08	67.90	57.06	72.37

BP: band power, GPFD: Grssberger and Procaccia-based fractal dimension, CSP: common spatial pattern, FBCSP: filter-bank CSP, KEFP-CSP: kernel eigen-FB CSP, PCA: principal component analysis.

**Table 5:** The Voting-based LOPO-CV Procedure.

Initialize $n_{t}$ Initialize free parameter(s) of the chosen method Initialize vote=0;
*Step 1*: EEG data of the first $n_{t}$ trials of the *i*th participant were used as the test data, and the EEG data of the first $n_{t}$ trials of the remaining participants ($n_{t}\times 23$ EEG data in total) were used for training the method (KEFB-CSP + classifier), where $1\leq n_{t}\leq 54$. *Step 2*: Fed the $n_{t}$ test data into the trained method, one test data at a time. Then, calculated the correct ratio for the *i*th participant. *Step 3*: If ratio>0.5, then vote=vote+1. *Step 4*: Repeated step 1 to 3 until every participant was chosen as the testing participant once. *Step 5*: Calculated the individual classification accuracy by CA=vote/24.

**Table 6:** The voting-based LOPO-CV results using the EEG signals of the first 15 trials ($n_{t}=15$), KEFB-CSP feature, and SVM classifier.

Participant	Classified as D	Classified as H	Correct Ratio	Participant	Classified as D	Classifier as H	Correct Ratio
**1 (D)**	15	0	1	**13 (H)**	4	11	0.73
**2 (D)**	15	0	1	**14 (H)**	5	10	0.67
**3 (D)**	15	0	1	**15 (H)**	9	5	0.33
**4 (D)**	0	15	0	**16 (H)**	4	12	0.8
**5 (D)**	15	0	1	**17 (H)**	0	15	1
**6 (D)**	6	9	0.4	**18 (H)**	4	11	0.73
**7 (D)**	11	4	0.73	**19 (H)**	0	15	1
**8 (D)**	15	0	1	**20 (H)**	0	15	1
**9 (D)**	15	0	1	**21 (H)**	15	0	0
**10 (D)**	15	0	1	**22 (H)**	0	15	1
**11 (D)**	9	6	0.6	**23 (H)**	0	15	1
**12 (D)**	15	0	1	**24 (H)**	0	15	1
Number of correctly classified patients			10	Number of correctly classified controls			10
Sensitivity = 83.33 % (10/12)				Specificity = 83.33 % (10/12)			
Individual classification accuracy = 83.33 % (20/24)							

D: depressed patients, H: healthy control.

**Table 7:** The voting-based LOPO-CV results using the EEG signal of the first trial ($n_{t}=1$), KEFB-CSP feature, and SVM classifier.

Participant	Classified as D	Classified as H	Correct Ratio	Participant	Classified as D	Classifier as H	Correct Ratio
**1 (D)**	1	0	1	**13 (H)**	0	1	1
**2 (D)**	1	0	1	**14 (H)**	0	1	1
**3 (D)**	1	0	1	**15 (H)**	1	0	0
**4 (D)**	1	0	1	**16 (H)**	0	1	1
**5 (D)**	1	0	1	**17 (H)**	0	1	1
**6 (D)**	1	0	1	**18 (H)**	1	0	0
**7 (D)**	1	0	1	**19 (H)**	0	1	1
**8 (D)**	1	0	1	**20 (H)**	0	1	1
**9 (D)**	1	0	1	**21 (H)**	0	1	1
**10 (D)**	1	0	1	**22 (H)**	0	1	1
**11 (D)**	1	0	1	**23 (H)**	0	1	1
**12 (D)**	1	0	1	**24 (H)**	0	1	1
Number of correctly classified patients			12	Number of correctly classified controls			10
Sensitivity = 100% (12/12)				Specificity = 83.33 % (10/12)			
Individual classification accuracy = 91.67 % (22/24)							

D: depressed patients, H: healthy control.

**Table 8:** Optimum Parameters of KEFB-CSP and SVM for Different Number of Trials $n_{t}$.

Methods and Parameters		1	3	7	11	15	19	23	27	54
KEFB-CSP	p	3	3	3	3	3	3	3	3	3
	σ	28	11	15	24	16	28	16	28	60
	*d*	5	2	2	1	4	1	3	3	3
SVM	$\sigma_{s}$	0.4	0.1	21	3.5	1.5	2	4.5	5.3	4
	*C*	100	100	100	100	100	100	50	50	100
